# Characterization of T cell phenotype and function in a double transgenic (collagen-specific TCR/HLA-DR1) humanized model of arthritis

**DOI:** 10.1186/ar4433

**Published:** 2014-01-10

**Authors:** Bo Tang, Seunghyun Kim, Sarah Hammond, David L Cullins, David D Brand, Edward F Rosloniec, John M Stuart, Arnold E Postlethwaite, Andrew H Kang, Linda K Myers

**Affiliations:** 1 Departments of Medicine, University of Tennessee Health Science Center, Memphis, TN 38163, USA; 2 Departments of Pediatrics, University of Tennessee Health Science Center, Memphis, TN 38163, USA; 3 Research Service, Veterans Affairs Medical Center, Memphis, TN 38104, USA; 4 Division of Rheumatology, 956 Court Avenue, Room G326, Memphis, TN 38163, USA

## Abstract

**Introduction:**

T cells orchestrate joint inflammation in rheumatoid arthritis (RA), yet they are difficult to study due to the small numbers of antigen-specific cells. The goal of this study was to characterize a new humanized model of autoimmune arthritis and to describe the phenotypic and functional changes that occur in autoimmune T cells following the induction of pathological events.

**Methods:**

We developed a double transgenic mouse containing both the HLA-DR1 transgene and an HLA-DR1-restricted collagen-specific TCR in order to obtain large numbers of antigen-specific T cells that can be used for immunologic studies.

**Results:**

*In vitro*, CII-specific T cells from this mouse proliferated vigorously in response to the CII immunodominant peptide A2 and the cells altered their phenotype to become predominately CD62L^low^ and CD44^high^ “activated” T cells. The response was accompanied by the production of Th1, Th2, and Th17-type cytokines. Following immunization with bovine CII/CFA, these mice develop an accelerated arthritis compared to single transgenic HLA-DR1 mice. On the other hand, when the mice were treated orally with the analog peptide A12, (a suppressive analog of collagen we have previously described), arthritis was significantly suppressed, despite the fact that >90% of the CD4+ T cells express the TCR Tg. In GALT tissues taken from the A12-treated mice, IL-2, IFN-γ, and IL-17 production to the autoimmune collagen determinant dropped while high levels of IL-10 and IL-4 were produced.

**Conclusions:**

We have developed a humanized model of autoimmune arthritis that will be useful for the study of T cell directed therapies as well as T cell mediated mechanisms of autoimmune diseases.

## Introduction

Although the majority of autoreactive T cells are deleted in the thymus, it is clear that some escape thymic selection, and once in the periphery are capable of mediating an autoimmune response. However, the precise mechanism by which T cell function is altered so that autoimmune diseases can develop is poorly understood. Some of the difficulty in deciphering these mechanisms is that the frequency of the Ag-specific T cells to be targeted is very limited, estimated to be 10^4^ or less [1]. Here, we describe the development of a double-transgenic mouse that expresses an autoreactive T cell receptor (TCR) specific for type II collagen (CII) and the human leukocyte antigen (HLA) -DRB1*0101, a haplotype susceptible to the induction of collagen-induced arthritis. These mice express a TCR specific for the HLA-DR1-restricted immunodominant determinant of CII, A2, on >90% of the CD4+ T cells. Despite the fact that they are capable of recognizing murine CII (mCII), they are not deleted in the thymus.

We have analyzed the function of these T cells using the collagen-induced arthritis model with the goal of determining the role that autoreactive T cells play in mediating autoimmune arthritis and in modulating the severity of the disease. We also investigated whether these autoreactive transgenic T cells could be manipulated to secrete primarily suppressive cytokines, thereby downregulating autoimmunity. We have treated these mice orally with an altered peptide ligand (APL) of collagen to determine how this therapy influences the cytokine responses of T cells located in murine gut-associated lymphatic tissue (GALT). We believe this transgenic (Tg) model can serve as an excellent vehicle for defining the molecular mechanisms by which T cells influence autoimmune arthritis and will help shape the development of future T-cell directed therapies for autoimmune diseases [2].

## Methods

### Preparation of tissue-derived CII and synthetic peptides

The following nomenclature is used to define the antigens used in this study: type II collagen, CII; bovine type II collagen, bCII; murine type II collagen, mCII; a peptide containing the immunodominant determinant sequence of both bovine and human CII (GIAGFKGEQGPKGEB), A2; a peptide containing the immunodominant determinant sequence of murine CII (GIAGFKGDQGPKGEB), mA2; a synthetic peptide representing the sequence (GIAGNKGDQGPKGEB), A12; the constituent protein chains of bovine CII isolated by carboxymethyl-cellulose chromatography, α1(II). B stands for 4-hydroxyproline.

Native type II collagen (CII) was solubilized from fetal calf articular cartilage or murine articular cartilage by limited pepsin-digestion and purified as described earlier [3]. The purified collagen was dissolved in cold 10 mM acetic acid at 4 mg/ml and stored frozen at -70°C until used. In some experiments denatured α1(II) chains were used. The synthetic peptides were supplied by Biomolecules Midwest Inc. (Waterloo, IL, USA).

### Animals

#### *Generation of Tg mice expressing DR*

Mice expressing the chimeric (human/mouse) DRB1*0101 construct were maintained in our onsite facility. The chimeric DRB1*0101 construct and the production of Tg mice expressing this construct have been previously described [4].

#### *Generation of double-transgenic (DR1-TCR) mice*

The CII-specific TCR-genes in the DR-transgenic mice, were expressed using the cassette vectors pT_α_cass and pT_β_cass provided by Benoist and Mathis [5]. Rearranged Vα2/Jα27 and Vβ8.1/D/Jβ2.4 fragments were obtained from a DR1-restricted A2-specific T cell hybridoma (E168) by rt-PCR. The PCR fragments were subcloned into the TCR cassette vectors, using the following modifications. For the α chain construct, a Xma I site was created 17 bp upstream of the ATG cordon of the TCR-Vα2/Jα27 cDNA; and an 11-bp sequence that represents the beginning of the J-C intron was added downstream of the Jα27 segment, followed by an artificial Not I site. The resulting cDNA fragment was introduced into the pT_α_cass between the Xma I and Not I sites to create the pT_α_ cass-Vα2. For the β chain construct, a Xho I site was created 12 bp upstream of the ATG cordon of Vβ8.1, and a 17 bp untranslated sequence representing the beginning of the J-C intron was introduced downstream of Jβ2.4, followed by a Sac II site. The resulting cDNA was introduced into the cassette vector pT_β_cass between the Xho I and Sac II sites to create pT_β_cass-Vβ8.1. The linearized DNA fragments (pT_α_cass-Vα2 and pT_β_cass-Vβ8.1) were simultaneously injected into fertilized eggs of FVB/N mice. Transgenic founders were identified by PCR analysis of tail DNA. Mice were backcrossed onto DR1-transgenic C57BL/10 mice to establish a TCR transgenic mouse line, and then backcrossed to the HLA-DR1 mice to develop double-transgenic mice. Mice used in these experiments were from generations N13 to N14 and were heterozygous for the TCR transgenes and homozygous for the HLA-DR1 transgene.

All mice were fed standard rodent chow (Ralston Purina Co., St Louis, MO, USA) and water *ad libitum*. Sentinel mice were routinely tested for murine pathogens. Experiments were conducted in accordance with approved Institutional Animal Care and Use Committee (IACUC) protocols. Mice were 8 to 12 weeks old.

#### *Antibodies*

The antibodies (Abs) used for flow cytometry included: peridinin chlorophyll protein (PerCP)-conjugated anti-CD4 (clone RM4-5), phycoerythrin (PE)-conjugated anti-CD8 (clone 53–6.7), PE-conjugated anti-CD62L (clone Mel-14), fluorescein isothiocyanate (FITC)-conjugated anti-V β8.1,2 (clone F23.1), APC-conjugated anti-CD44 (clone IM7), and FITC-conjugated anti-Vα2 (clone: B20.1). All Abs were purchased from BD PharMingen (San Diego, CA, USA) and used according to the manufacturer’s recommendations.

#### *Flow cytometry*

Splenocytes, peripheral blood cells, or inguinal lymph node cells from mice were isolated and the phenotype was determined by multiparameter flow cytometry using an LSRII flow cytometer (BD Biosciences, San Jose, CA, USA). Cells were labeled with fluorochrome antibodies specific for CD4, CD25, or CD44, (BD Biosciences). In some experiments intracellular labeling was performed using antibodies specific for FoxP3 (FJK-16 s, eBioscience, San Diego, CA, USA). In the experiments involving intracellular staining, gating was performed on both CD4+ and Vβ8+ cells. A minimum of 10,000 cells was analyzed from each sample and the final analysis was performed using FlowJo software (Tree Star, Ashland, OR, USA).

#### *Proliferation assays*

Spleen cells were cultured with various concentrations of collagen and collagen peptides in 96-well plates at 4.5 × 10^5^/well in 300 μl of HL-1 medium supplemented with 50 μM of 2-ME and 0.1% BSA (fraction V, Gig free, low end toxin; Sigma-Aldrich, St Louis, MO, USA) at 37°C, 5% humidified CO_2_ for 4 days. Eighteen hours before the termination of the cultures, 1 μCi of (^3^H)thymidine (New England Nuclear, Boston, MA, USA) was added to each well. Cells were harvested onto glass-fiber filters and counted on a Matrix 96 direct ionization beta counter (Packard Instrument, Meriden, CT, USA). Results were confirmed by replicate experiments and all data are expressed as disintegrations per minute (dpm).

#### *Immunizations and arthritis induction*

Mice were immunized with bCII for the induction of arthritis. bCII was dissolved in 10 mM cold acetic acid. The bCII was then emulsified with CFA (Complete Freund’s Adjuvant) as previously described [3]. Mice were immunized subcutaneously at the base of the tail with 100 μg of bCII. For other immunizations, either peptide A12 or Ova emulsified with CFA was given subcutaneously so that each mouse received 100 μg of protein.

#### *Measurement of the severity of arthritis*

The severity of arthritis was determined by visually examining each forepaw and hindpaw and scoring them on a scale of 0 to 4 as described previously [3]. Scoring was conducted by two examiners, one of whom was unaware of the identity of the treatment groups. Each mouse was scored thrice weekly beginning 3 weeks post immunization and continuing for 8 weeks. The mean severity score (sum of the severity scores for the group on each day/total number of animals in the group) was recorded at each time point.

#### *Measurement of serum antibody titers*

Mice were bled at 4 weeks after immunization and sera were analyzed for antibodies reactive with native murine CII using a previously described modification of an ELISA [3]. Serial dilutions of a standard serum were added to each plate. From these values, a standard curve was derived by computer analysis using a four-parameter logistic curve. Results are reported as units of activity, derived by comparison of test sera with the curve derived from the standard serum which was arbitrarily defined as having 50 units of activity. Reactivity to mCII was not detected in sera obtained from normal mice.

#### *Adoptive transfer experiments*

Inguinal lymph-node cells from DR1-TCR double-transgenic mice previously immunized with either A12 or Ova in CFA were collected 8 days after immunization and the CD4+ cell subset was fractionated using ferromagnetic beads (Miltenyi Biotec, Gladbach, Germany) according to the manufacturer's protocol. The purity of the resulting cell population was confirmed by flow cytometry to have >95% purity. Recipient mice were given cells intravenously and received 5 × 10^5^ cells. All recipient mice were immunized with bCII on the day of the cell transfer and observed for arthritis.

#### *Oral treatment with peptide*

For the study of events following oral administration of the peptide A12 in the DR1-TCR Tg mice, either A12 or Ova was dissolved in PBS. DR1-TCR mice were fed doses of 10, 50, or 100 μg A12, or 50 μg Ova by oral gavages three times a week. The treatments began either the day after immunization of mice with bCII/CFA (prevention protocol) or the day of the onset of arthritis (treatment protocol) and continued for the duration of the experiment.

#### *Measurement of cytokines*

To measure cytokines, in some experiments, naïve splenocytes from DR1-TCR transgenic mice were cultured with bovine α1(II), A2, mA2, or A12. In other experiments, DR1-TCR transgenic and DR1 Tg mice were immunized with bCII/CFA and lymph-node cells were taken from the mice 10 days after immunization and were cultured with a peptide representing the immunodominant determinant of murine collagen (mA2) so that supernatants could be analyzed for the presence of cytokines. In a third set of experiments, DR1-TCR double-transgenic mice were treated with 8 doses of 50 μg of either A12 or PBS by oral gavage, sacrificed after the last gavage and GALT cell populations were removed. Single-cell suspensions of mesenteric lymph-node cells and Peyer’s patch cells were made and pooled prior to culture in 48-well plates in triplicates. Each well contained 500 μl single cell suspension (5 × 10^5^/ml) + indicated concentrations of each peptide, or PBS. Media used were X-VIVO 10 (Biowhittaker, Radnor, PA) for two-day cultures or DMEM-complete (DMEM + 1% Pen-strep, 1% 2-ME, 1% glutamax, 1% sodium pyruvate, 10% FBS) for 6-day cultures. Cells were incubated at 37°C and harvested after 72 hours.

Each culture was set up using cells from three mice run in triplicates and supernatants were analyzed for the presence of IL-10, IL-4, IFN-γ, and IL-17A using a Bio-plex mouse cytokine assay (Bio-Rad, Hercules, CA, USA) according to the manufacturer’s protocol. Values are expressed as picograms per ml and represent the mean values for each group taken from three separate experiments.

### Statistical analysis

Mean severity scores, antibody titers, and cytokine levels were compared using the Mann–Whitney test. The numbers of arthritic limbs were compared using Fischer’s exact test.

## Results

### Generation of the double-transgenic mice

**Figure 1:**
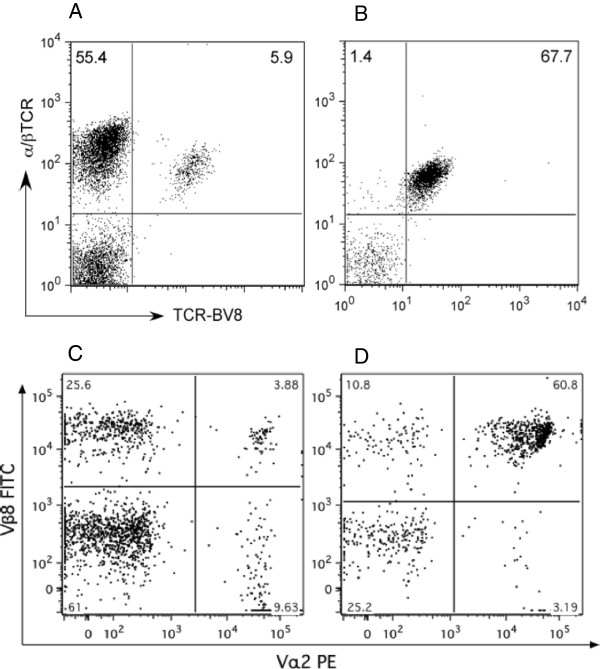
**Development of a double-transgenic DR1-T cell receptor (TCR) Tg mouse model of autoimmune arthritis.** The double-transgenic DR1-TCR Tg mouse model was developed and backcrossed onto DR1 transgenic mice as described in Methods. To detect the presence of the transgene, spleen cells were stained with a combination of anti-Vβ8- fluorescein isothiocyanate (FITC) and anti- TCR-phycoerythrin (PE). **(A)** Representative data obtained from the single-transgenic DR1 mouse; **(B)** representative data obtained from the double-transgenic mouse. In a similar manner, peripheral blood cells were obtained from the single-transgenic DR1 mouse **(C)** and compared to cells from the double-transgenic mouse **(D)**, staining with antibodies specific for CD4+, Vβ8.1 and Vα2. The majority of the CD4+ cells in the double-transgenic mouse express both Vβ8.1 and Vα2. Data are based on the analysis of 10,000 gated events with the gate set on forward versus side scatter to exclude non-lymphoid cells and dead cells.

To study the role that T cells play in a milieu that contains a human transgene (HLA DRB1*0101) that correlates with susceptibility to human rheumatoid arthritis (RA), a double-transgenic (DR1-TCR) Tg mouse was developed. The cDNA encoding an A2-specific TCR was cloned by RT-PCR from a DR1-restricted T cell hybridoma that recognizes both bCII (immunogen) and mCII (autoantigen). This TCR uses *V*α*2-J*α27 gene segments and *V*β*8.1 J*β*2.4* gene segments similar to other CII-specific DR1-restricted TCR previously described [6,7]. The Vα and Vβ cDNA were cloned into the plasmid pT_α_cass and pT_β_cass developed by Mathis and Benoit [5]. Following co-injection, founders were identified by PCR and immunofluorescence and backcrossed to mice bearing the DR1 transgene [4]. As shown in Figure 1, the *V*β*8* transgene was expressed by >90% of the α/βTCR T cell population of the spleen (Figure 1B), which is significantly higher than the 9% of the α/βTCR + cells that express the endogenous Vβ8 in the non-Tg littermates (Figure 1A). Similarly, when examining peripheral blood cells, the TCR Vα2 and *V*β*8* are co-expressed on the majority of the CD4+ T cells from the double-transgenic mice (panel D) compared to cells from the DR1 single-transgenic mice (Figure 1C).

### Phenotype of TCR T cells

**Figure 2:**
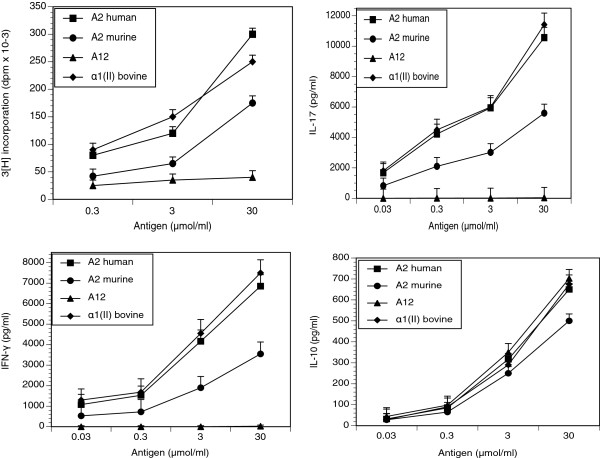
**Naive spleen cells from DR1-T cell receptor (TCR) Tg mice respond to culture with type II collagen (CII).** Spleen cells from naive DR1-TCR Tg mice were cultured with human A2, murine A2, A12 or bovine α1(II) chains with titrated doses. Cytokines are expressed as pg/ml. Proliferation was measured by incorporation of (3H)-thymidine and is expressed as the mean disintegrations per minute (dpm) of triplicate cultures. Data are expressed as means ± standard error of the mean of experiments using three separate mice. Responses to the murine determinant differ significantly from those induced to either human A2 or bovine α1(II) when comparing proliferation, IFN-γ, IL-17, or IL-10 (*P* ≤0.05 using the Mann–Whitney test). Responses to A12 differ significantly from the responses to human A2, α 1(II) and murine A2 in proliferation, IFN- γ and IL-17 (*P* ≤0.05 using the Mann–Whitney test) but the A12-induced IL-10 response was not different.

The TCR Tg is fully functional as measured by the ability of the T cells to proliferate specifically in response to peptide presentation by DR1. When Tg T cells were stimulated *in vitro* with either bovine α1(II) or A2, the cells proliferated vigorously and induced a full array of cytokines (IFN-γ, IL-17, IL-10) in the presence of antigen presenting cells (APCs) (Figure 2). No proliferative response to Ova was observed and T cells from non-Tg littermates did not proliferate (data not shown). Moreover, we demonstrated that these T cells are cross-reactive with mA2, demonstrating both proliferation and a full array of cytokines, although these responses were weaker than those induced by A2 (Figure 2). These data reflect our prior observation that changing the Asp (A2) at residue 266 to Glu (mA2), which is the residue that interacts with the P4 binding pocket of the HLA-DR1, causes a lower affinity of binding to the DR1, inducing a weaker response from the transgenic T cells compared to that induced by A2 [8]. On the other hand, the A12 peptide, which contains amino acid substitutions at positions 263 (N) and 266 (D) so that interaction with both the P1 and P4 binding pockets of the DR1 are more profoundly disrupted, induces a significant IL-10 response from the transgenic T cells (Figure 2) unaccompanied by proliferative or inflammatory cytokine responses.

In order to compare autoreactive T cell responses from the double-transgenic T cells with those from the single-transgenic DR1 mice, we immunized mice with bCII and cultured the lymph-node T cells with the mA2 peptide in the presence of APCs (Table 1). The resulting supernatants demonstrated a vigorous production of T helper (Th)1, Th2, and Th17 cytokines that were greater than those induced by T cells obtained from CII-immunized single-transgenic DR1 mice. The differences were most striking in the inflammatory profiles (Table 1). Taken together, these data demonstrate that these double-transgenic T cells recognize the primary autoantigenic determinants of murine CII and the exaggerated responses reflect the presence of a large number of fully functional CII-reactive T cells.

**Table 1:** Cytokines produced in response to murine collagen

**Mouse**	**Culture**		**Cytokines (pg/ml)**	
		**IFN-γ**	**IL-17**	**IL-4**
DR1/TCRtg	No Ag (antigen)	0	17 ± 5	0
	mA2 (3 μmol/ml)	13,522 ± 154	9,849 ± 255	297 ± 24
	mA2 (0.3 μmol/ml)	5,167 ± 95	5,206 ± 175	142 ± 19
DR1	No Ag (antigen)	59 ± 11	184 ± 43	23 ± 12
	mA2 (3 μmol/ml)	1,116 ± 22*	1,082 ± 155*	85 ± 18*
	mA2 (0.3 μmol/ml)	737 ± 38*	695 ± 29*	42 ± 11*

Inguinal lymph-node cells from type II collagen (CII)-immunized DR1 or DR1-T cell receptor (TCR) Tg mice were taken from the mice 10 days after immunization and were cultured with peptide mA2 so that supernatants could be analyzed for the presence of cytokines. The numbers indicated represent the means ± standard error of the mean of three separate experiments. When cultures from DR1/TCR tg mice were compared with those from DR1 mice, the cytokine responses to collagen were all elevated, T helper (Th)1, Th2, and Th17. *P ≤0.01 when mA2 responses from DR1 mice were compared with mA2 responses from DR1-TCR/tg mice. *Ag*, antigen; *mA2*, murine A2 peptide.

**Figure 3:**
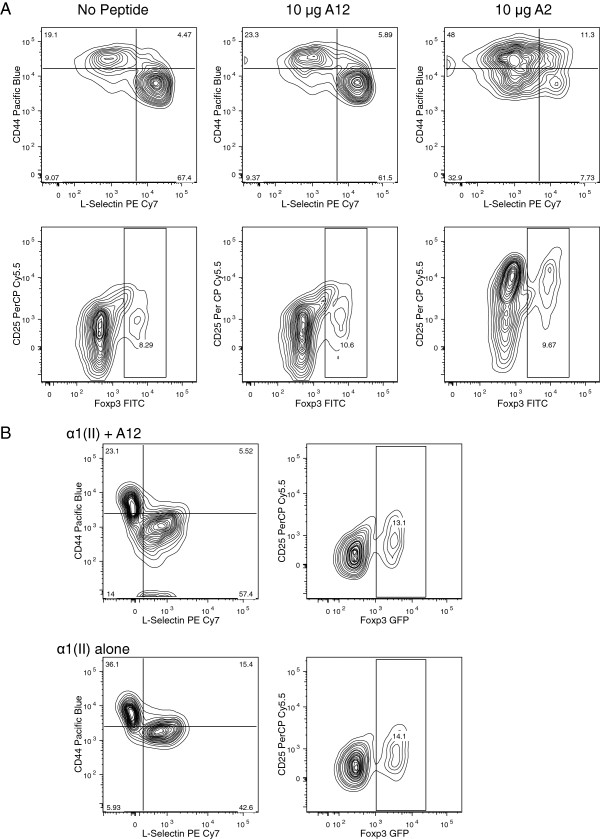
**Phenotype of splenocytes from double-transgenic mice cultured with peptides. (A)** CD4+ splenocytes from double-transgenic mice cultured with peptide A2, developed a CD62L^hi^ CD62L^lo^ memory phenotype, whereas CD4+ cultured with A12 peptide or media alone did not change (%CD44^hi^CD62L^lo^CD4+ T cells = 18 ± 3 for No Ag, 23 ± 5 for A12, and 47 ± 6 for A2; *P* ≤0.002 for A2 versus No Ag and *P* ≤0.006 for A2 versus A12). Splenocytes from the double-transgenic mouse cultured with peptide A2 or A12 had no significant differences in numbers of Treg cells (%CD25^hi^Foxp3^hi^CD4+ T cells = 8 ± 2 for No Ag, 9 ± 2 for A12, and 9 ± 3 for A2). **(B)** Phenotype of splenocytes from double-transgenic mice tested directly *ex vivo*: α1(II) was administered *in vivo* (100 μg/mouse intravenously) alone, or with 100 μg peptide A12. Three days later, CD4+ T cells treated with α1(II) developed a CD62L^hi^ CD62L^lo^ memory phenotype, whereas A12 peptide significantly decreased expression of activation markers (%CD44^hi^CD62L^lo^CD4+ T cells = 22 ± 7 for A12 + α1(II)-treated mice, and 37 ± 6 for α1(II)-treated mice; *P* ≤0.02 for α1(II)-treated versus α1(II) + A12 treated). Staining for intracellular Foxp3 was not different between groups tested directly *ex vivo* (15 ± 7 for α1(II)-treated mice versus 13 ± 5 for α1(II) + A12-treated mice. Negative control (naïve double-transgenic T cells) had the pattern: (%CD44^hi^CD62L^lo^CD4+ T cells = 16 ± 4 and Foxp3 + CD4 T cells = 12 ± 5). Data representative of three separate experiments (**A** and **B**).

To evaluate phenotypic changes, CD4+ splenocytes were cultured with A2 in the presence of APCs and tested for activation and memory-marker expression (Figure 3A). These analyses revealed that as early as 24 h post culture, the expression levels of two cell-surface markers associated with the activation/memory phenotypes, CD44high and CD62Llow, underwent marked shifts. The vast majority of the A2-cultured cells now expressed CD44high and CD62low compared to cells cultured with a control analog peptide A12 or cells cultured with media alone. Only, 9.7 percent of the T cells cultured with A2 expressed the Treg-associated nuclear transcription factor Foxp3, similar to the percentages of Tregs identified in cultures of cells incubated with media alone. Thus, using this model, we have demonstrated that challenge with peptide A2 induces the majority of the T cells to enter a period of activation, acquiring a memory phenotype.

Similarly, when α1(II) was administered *in vivo* to the double transgenic mice, the CD4+ T cells tested directly *ex vivo* were found to have increased numbers of activated CD44^hi^ and CD62L^lo^ CD4+ cells (Figure 3B). On the other hand, the addition of the A12 peptide significantly decreased the expression of activation markers. Staining for intracellular Foxp3 did not reveal differences between the two groups tested directly *ex vivo* (Figure 3B).

### Induction of autoantibodies and disease in DR1-TCR Tg mice

**Figure 4:**
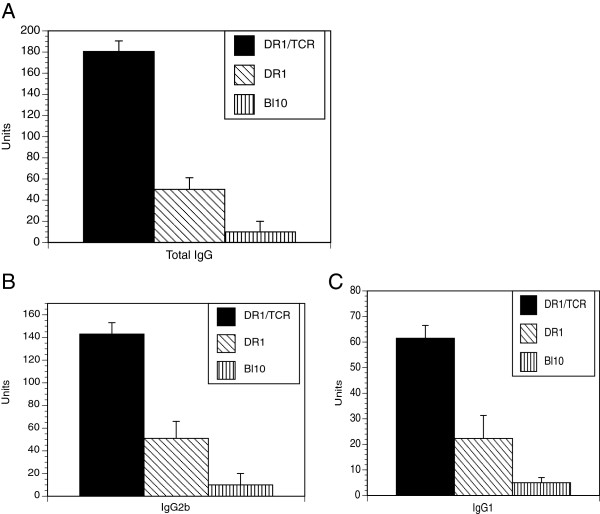
**Production of antibody (Ab) specific for murine type II collagen (mCII) by double DR1-T cell receptor (TCR) Tg and DR1 Tg mice.** Double- and single-transgenic littermates were immunized with 100 μg of bCII emulsified in CFA (Complete Freund’s Adjuvant) as described in Methods. Serum samples were collected 27 days after immunization and analyzed for quantity of mCII-specific Ab by ELISA. Units of Ab were calculated using standard reference sera. Data are expressed as mean titers of 10 mice per group ± standard error of the mean (total IgG titers, double-transgenic versus single-transgenic, 180.5 ± 34 versus 50.2 ± 11, *P* = 0.001; IgG1 titers, double-transgenic versus single-transgenic: 61.5 ± 22.3 versus 12.3 ± 6.1, *P* = 0.007; IgG2b titers 143.45.9 versus 51.3 ± 16.6, *P* = 0.0008; IgG3 titers 13.1 ± 5.6 versus 14.2 ± 3.2, *P* = 0.69).

**Figure 5:**
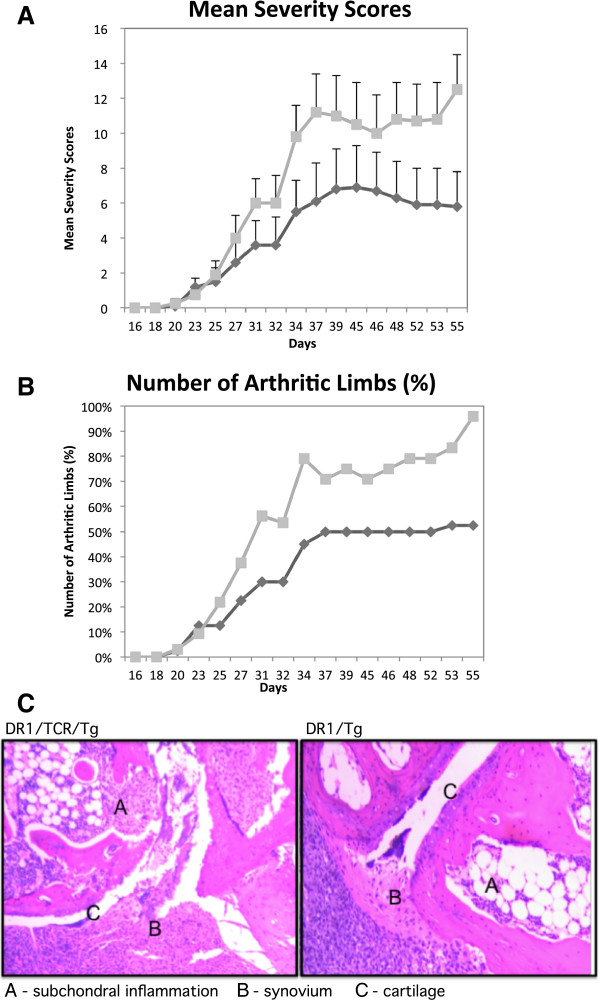
**Disease severity in DR1-T cell receptor (TCR) Tg mice (panels A and B).** The severity (panel **A**) and percentage of arthritic limbs (panel **B**) of disease in double-transgenic mice (squares, n = 13) compared with DR1 Tg littermate controls (diamonds, n = 15). All animals were challenged with a dose of 100 μg of bovine type II collagen (bCII) emulsified in CFA (Complete Freund’s Adjuvant) for the induction of disease. Mice were scored for arthritis severity as described in Methods. Panel **A**, *P* = 0.009 on day 55 when comparing the mean severity scores ± standard error of the mean using the Mann–Whitney test; panel **B**, *P* = 0.002 on day 55 when comparing the number of arthritic limbs using Fischer’s exact test. The final incidence was 70% for the single-transgenic mice and 90% for the double-transgenic mice. Histology of joints of arthritic animals (panel **C**): left, joint from double-transgenic animal showing more eroding pannus and greater amounts of damaged articular cartilage compared to a representative joint (right), from a single-transgenic animal. The animals were sacrificed eight weeks after immunization. Joints were collected, fixed with formaldehyde, and decalcified prior to staining with H&E. The tissues were photographed using an inverted-phase contrast microscope (original magnification: 50×). The data shown are representative of data obtained by analyzing numerous sections from hindpaws taken from 6 animals per group.

When the Tg mice were immunized with bCII/CFA, they developed a strong T cell-dependent Ab response to the autoantigen, mCII (Figure 4), greater than that elicited in control mice bearing only the DR1 transgene The increases were pronounced in both immunoglobulin (Ig)G2 and IgG1 subsets. Moreover, offspring from the double-transgenic founders developed a more severe form of disease as compared with the DR1 littermates, as measured by both severity scores and the number of arthritic limbs (Figure 5, panels A and B). Although both groups of mice developed arthritis around 20 days after immunization and continued to progress in severity until the end of the experiment (day 55), the severity scores were markedly different (*P* = 0.009). Histology confirmed that the double-transgenic mice had greater pannus formation and cartilage destruction when compared to arthritic DR1 mice (Figure 5, panel C). We have not observed spontaneous arthritis in the double-transgenic mice up to 24 months of age, despite large numbers of CII-specific T cells.

### Induction of suppression of arthritis

**Figure 6:**
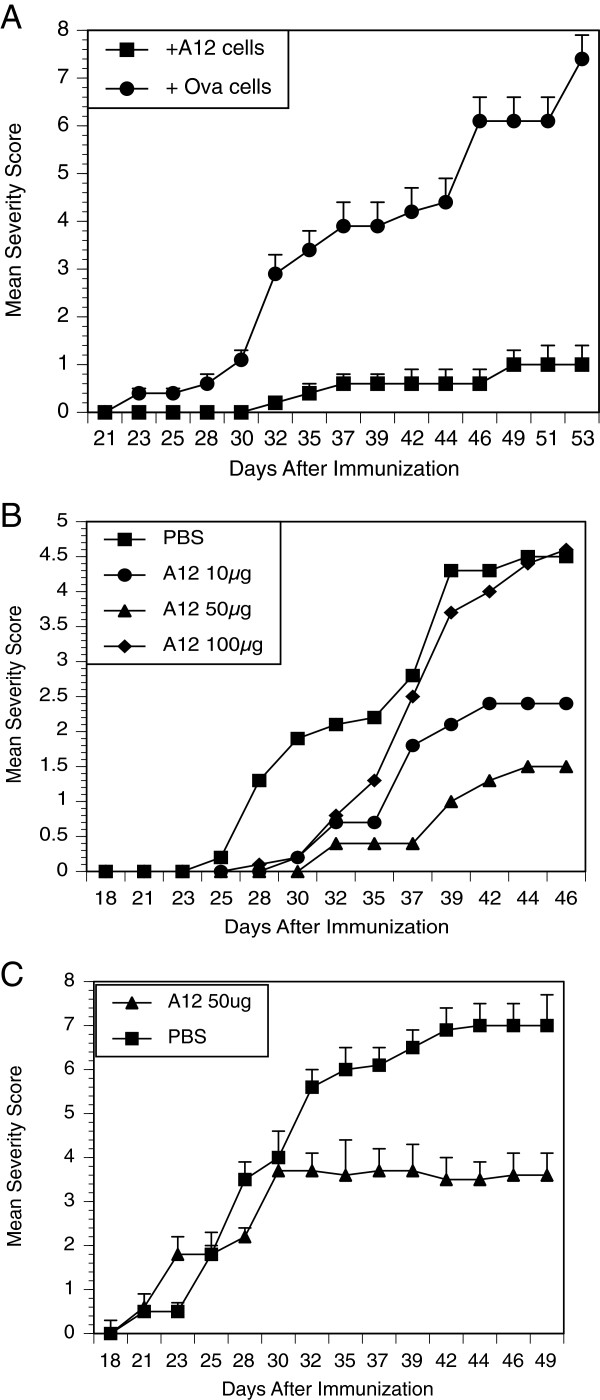
**Treatment with either A12-immune cells or A12 peptide can suppress arthritis.** Panel **A**: CD4+ T cells from the double transgenic mice treated with A12 can transfer the suppression of arthritis. Single-transgenic DR1 mice (10/group) were infused with 5 × 10^5^ cells of A12-primed CD4+ T cells from double-transgenic mice or Ova-primed CD4+ T cells from single-transgenic DR1 mice. Recipient animals were immunized with bovine type II collagen (bCII)/CFA and observed for arthritis. Only A12-immune cells prevented collagen-induced arthritis (final severity scores 1.0 ± 0.5 versus 7.4 ± 1.2, *P* ≤0.0001; final incidence 90% versus 10% in the treatment group). (Panel **B**) Oral treatment with peptide A12 (prevention protocol). Groups of 12 double-transgenic mice were administered PBS or A12 peptide (oral gavage three times/week), beginning the day after immunization with bCII/CFA, continuing for the duration of the experiment. On day 46, mice fed PBS had a severity score of 4.5 ± 0.4, which differed from mice fed 10 μg of A12 (2.4 ± 0.5, *P* ≤0.05) and mice fed 50 μg of A12 (1.5 ± 0.04, *P* ≤0.01; final incidence = 80% in the control and 20% in the 50-μg group). (Panel **C**) Oral treatment with peptide A12 (treatment protocol). Groups of 10 double-transgenic mice were administered PBS or A12 peptide (50 μg/dose, oral gavage three times/week) beginning the day of arthritis onset. On day 46, mice fed PBS had a severity score of 6.8 ± 0.6, which differed from mice fed 50 μg of A12 (3.6 ± 0.4, *P* ≤0.05) (Mann–Whitney test). (Final incidence = 90% in controls and 60% in the treatment group).

Based on the overwhelming T cell response and accelerated arthritis development, we wondered whether it would be possible to suppress the severity of the autoimmune arthritis. To resolve whether inflammatory T cells from the double-transgenic mice could successfully be shut down or redirected, we treated them with an altered peptide ligand of CII, A12, which we have previously described, and performed adoptive-transfer experiments [9]. Draining inguinal lymph-node cells from DR1-TCR Tg mice previously immunized with A12/ CFA or DR1 mice immunized with OVA/CFA alone were collected and CD4+ cells were fractionated using ferromagnetic beads. Either A12-immune or control CD4+ T cells were infused intravenously into DR1 tg mice prior to immunization with bCII/CFA, so that mice could be observed for effects on collagen-induced arthritis. When infused prior to the induction of arthritis, we observed (Figure 6, panel A), that the A12-immune T cells from the double-transgenic mice were extremely effective in suppressing arthritis, compared to control cells. Mean antibody titers to murine CII taken six weeks after immunization were decreased in mice given the A12-immune T cells, compared to mice given CD4+ control T cells (52.2 ± 16 in mice given control cells versus 11 ± 7 in mice given A12-immune cell, *P* ≤0.005).

### Treatment of arthritis

Our priority, however, was to establish whether arthritis could be suppressed in the double-transgenic mice once the immune response had been established. We elected to use the oral route of administration, a route more easily translatable into human usage. Therefore, when double-transgenic mice were treated orally with A12 in a preventive protocol (Figure 6, panel B), we found that A12 significantly reduced the severity of arthritis in the double-Tg mice. The mice treated with OVA developed arthritis at an expected accelerated rate, whereas by the A12-treated mice had significantly less severe disease. Interestingly, the dose response exhibited a bell-shaped curve with the 50 μg dose having the greatest effect on arthritis with protection afforded for the duration of the treatment. Mice treated with ether 10 μg or 100 μg had less suppression (Figure 6, panel B). In another set of experiments, mice were treated with the A12 peptide using a treatment protocol. Mice were fed daily doses of either PBS or 50 μg if A12, beginning the day of the onset of arthritis (Figure 6, panel C). Again, oral treatment with A12 significantly attenuated the severity of arthritis.

### Cytokine analysis

The GALT is constituted of a wide variety of cells, which are often grouped in complex structures, such as Peyer’s patches, mesenteric lymph nodes or isolated lymphoid follicles, or spread throughout the lamina propria. As orally ingested substances invariably reach the intestines, are absorbed by the organism and sampled by the immune system, pooled cells from Peyer’s patches and mesenteric lymph-node cells were recovered directly *ex vivo*, and tested for cytokine production following oral treatment with A12. When GALT cells were recovered from mice given A12 orally in the prevention protocol and cultured with mA2 the supernatants revealed an enhanced production of the suppressive cytokines IL-10 and IL-4 (Table 2). In contrast, IFN-γ and IL-17 production was significantly reduced compared to mice that were treated with PBS (Table 2). Collectively, the *in vivo* and *in vitro* experiments confirm that the suppression of arthritis by A12-specific T cells involves active secretion of suppressive cytokines resulting in downregulation of the inflammatory cytokines IL17 and IFN-γ.

**Table 2:** Cytokine responses from mice treated with oral A12

**Treatment**	**Culture**	**Cytokines (pg/ml)**			
		**IFN-γ**	**IL-17**	**IL-10**	**IL-4**
PBS	No Ag	10 ± 7	17 ± 8	17 ± 10	5 ± 3
	mA2	280 ± 22	256 ± 28	45 ± 14	25 ± 6
A12	No Ag	2 ± 3	2 ± 1	15 ± 9	4 ± 3
	mA2	12 ± 20*	2 ± 2*	105 ± 18*	48 ± 8*

Pooled cells from Peyer’s patches and mesenteric lymph nodes from A12-treated DR1/T cell receptor (TCR) Tg mice were taken from the mice 10 days after treatment with oral gavage was completed, and were cultured with murine A2 peptide (mA2) (3 μmol/ml) so that supernatants could be analyzed for the presence of cytokines. When cultures from mice treated with A12 were compared with those from mice treated with PBS alone, the IL-10 response to mA2 was elevated whereas the inflammatory cytokines (IFN-γ and IL-17), were suppressed. The numbers indicated represent the means ± standard error of the mean of three separate experiments. *P ≤0.01 when type II collagen (CII) responses from A12-treated mice were compared with CII responses from untreated mice.

## Discussion

One of the key roles of the immune system is to protect the organism against infectious diseases and cancer, while avoiding autoimmune damage. Although T cell responses are expected to be tightly regulated to prevent T cell hyperactivation, which may lead to autoimmune pathology, our data clearly demonstrate that autoreactive T cells can be activated and induced to secrete inflammatory cytokines, causing significant tissue damage, and playing a major role in increasing the severity of autoimmune arthritis. Although others have previously observed that T cells play a critical role in autoimmunity in other animal models, that is, proteoglycan (PG)-induced arthritis (PGIA) and K/BxN spontaneous arthritis [10] as well as collagen-induced arthritis (CIA) [11], the ability to make double-transgenic mice that have a large number of T cells restricted by the common RA-susceptible major histocompatibility complex (MHC) molecule, DRB*0101 (DR1), allows greater ease and clarity in investigating the mechanisms by which T cells become dysregulated during the initiation of arthritis or how their behavior is altered when exposed to new therapies.

We demonstrated that in the CIA autoimmune arthritis model, immunization with bCII/CFA causes an accelerated and more severe arthritis compared to mice bearing only the HLA-DR1 transgene. *In vitro*, bCII-specific double-transgenic T cells readily demonstrate both proliferation and production of Th1, Th2, and Th17-type cytokines when challenged with the A2 peptide, reflecting the presence of a large number of fully functional CII-reactive T cells. Using this model, we have confirmed that activation of a large number of DR1-restricted autoimmune CD4+ T cells enhances the severity of the arthritis, and this phenomenon is associated with both increased numbers of autoantibodies and enhanced levels of inflammatory cytokines. These data reflect the concept that T-cell recognition of a systemically expressed self-peptide can lead to more severe disease, and that inflammatory CD4 + T-cells are dysregulated in autoimmune arthritis [12-15].

On the other hand, an understanding of the mechanisms by which T cells can be manipulated to control disease is critical if T cell-specific therapies are to be successfully implemented. CD4+ helper T cells differentiate into specialized subsets, which attain restricted patterns of cytokine secretion and specific expression of master transcription factors in response to pathogens [16]. However, some of the distinctions between these groups are beginning to blur. Therefore, it becomes imperative to clearly define the functional and phenotypic attributes of autoreactive cells during the induction of suppression.

We have used the new humanized double-transgenic model of arthritis to test oral administration of the altered peptide ligand A12 peptide and to demonstrate that T cells found in the GALT tissues release IL-10 and IL-4 in response to a murine autoantigen [17]. Our therapy is based on our previous discovery that A12 could profoundly suppress arthritis in the CIA model by inducing a unique inhibitory T cell [18]. When the A12 peptide was administered orally to double-transgenic mice, arthritis was significantly suppressed, despite the fact that >90% of the CD4+ T cells express the Tg. The IL-2, IFN-γ, and IL-17 production dropped and was replaced by IL-10 and IL-4. The response to A12 was dose dependent, with low doses being more effective than high doses. We believe that the bell-shaped curve induced by oral A12, is consistent with antigen processing by the GALT, leading to T cell cytokine secretion. Other investigators have reported that proteins administered orally in low doses require the induction of both IL-4 and IL-10 to suppress disease and that this induction is consistent with processing by the GALT. High doses of antigens involve a different mechanism [19,20]. Our finding that A12 stimulates the secretion of IL-4 and IL-10 in GALT tissues, leading to suppression of arthritis, is consistent with evidence from other animal models, showing that release of IL-10 and IL-4 [17] inhibits inflammatory bowel disease [21], experimental autoimmune encephalomyelitis [22], and diabetes [17].

The success of the best-known altered peptide ligand (APL), Copaxone (glatiramer acetate) as a T cell-directed first-line therapy for multiple sclerosis (MS), suggests that peptide therapies based on naturally occurring proteins may provide an effective first-line alternative to biologic drugs for the treatment of arthritis and that animal models play a critical role in determining their efficacy and safety profiles. Although earlier clinical trials of glatiramer had conflicting results, two more recent trials have demonstrated its efficacy in treating MS, that is, the phase-3, randomized CONFIRM trial [23-26] and a large National Institutes of Health (NIH)-sponsored (CombiRx Trial) [27]. Its safety profile suggests that drugs based on APLs will have an excellent risk/benefit ratio [28].

We believe that this double-transgenic animal model of arthritis will be useful for the development and refinement of increasingly more sophisticated T cell-directed therapies for RA [29,30]. The difficulty of obtaining human GALT suggests that murine models will be invaluable in precisely delineating the role that T cells play when therapies are administered orally.

## Conclusions

This study provides a detailed description of the phenotype of a new double-transgenic (DR1, CII-specific TCR) mouse. We believe that this mouse will become an important tool to allow careful dissection of the role that T cells play in regulating autoimmune arthritis, and may shape new therapeutic approaches to autoimmune diseases. As several strategies for enhancing the efficacy of adoptive T cell therapy have been developed and introduced in clinical settings [31], we believe that this humanized arthritis model will be invaluable in advancing our understanding of the mechanisms that control the pathogenesis of RA.

## Abbreviations

A12: synthetic peptide representing the sequence (GIAGNKGDQGPKGEB); A2: peptide containing the immunodominant determinant sequence of bovine type II collagen (GIAGFKGEQGPKGEB); Abs: antibodies; APC: antigen-presenting cell; APL: altered peptide ligand; B: hydroxyproline; bCII: bovine type II collagen; bp: base pairs; BSA: bovine serum albumin; CIA: collagen-induced arthritis; CII: type II collagen; DMEM: Dulbecco's modified Eagle's medium; dpm: disintegrations per minute; ELISA: enzyme-linked immunosorbent assay; FBS: fetal bovine serum; FITC: fluorescein isothiocyanate; GALT: gut-associated lymphatic tissue; H&E: hematoxylin and eosin; HLA: human leukocyte antigen; IFN: interferon; Ig: immunoglobulin; IL: interleukin; mCII: murine type II collagen; MHC: major histocompatibility complex; MS: multiple sclerosis; murine A2: peptide containing the immunodominant determinant sequence of murine type II collagen (GIAGFKGDQGPKGEB); PBS: phosphate-buffered saline; PE: phycoerythrin; PerCP: peridinin chlorophyll protein; RA: rheumatoid arthritis; TCR: T cell receptor; Tg: transgene; Th: T helper; α1(II): constituent protein chains of bovine type II collagen isolated by carboxymethyl-cellulose chromatography.

## Competing interests

The authors have no competing interests.

## Authors’ contributions

All authors have made substantial contributions to the conception and design of the experiments. SK, SH, and DLC designed and carried out all the studies analyzing the T cells *in vitro*; BT, EFR, and AEP designed and carried out the studies involving the transgenic mice; DDB designed and participated in the studies using flow cytometry; LKM, JS, and AHK designed and participated in studies using the collagen-induced arthritis animal model. All authors were involved in drafting and revising the manuscript critically for important intellectual content. All authors have read and approved the final manuscript.
